# Presynaptic mechanisms of neuronal plasticity and their role in epilepsy

**DOI:** 10.3389/fncel.2014.00164

**Published:** 2014-06-17

**Authors:** Jochen C. Meier, Marcus Semtner, Aline Winkelmann, Jakob Wolfart

**Affiliations:** ^1^RNA Editing and Hyperexcitability Disorders, Max Delbrück Centre for Molecular Medicine, NeurosciencesBerlin, Germany; ^2^Oscar Langendorff Institute of Physiology, University of RostockRostock, Germany

**Keywords:** epilepsy, axon, RNA editing, potassium channels, glycine receptor, homeostatic regulation, neuropsychiatric disorders, hippocampus

## Abstract

Synaptic communication requires constant adjustments of pre- and postsynaptic efficacies. In addition to synaptic long term plasticity, the presynaptic machinery underlies homeostatic regulations which prevent out of range transmitter release. In this minireview we will discuss the relevance of selected presynaptic mechanisms to epilepsy including voltage- and ligand-gated ion channels as well as cannabinoid and adenosine receptor signaling.

## Introduction

Many studies highlighted the importance of homeostasis in neuronal signaling either within neurons, then called “intrinsic plasticity” or between neurons, referred to as “synaptic scaling” (Davis and Bezprozvanny, 2001; Eichler and Meier, 2008; Turrigiano, 2011). Classic synaptic plasticity in its non-homeostatic “Hebbian” form and pathological disturbances need counterbalancing homeostatic scaling mechanisms (Abbott and Nelson, 2000). The latter was mainly regarded from the postsynaptic perspective (Thiagarajan et al., 2005; Groth et al., 2011). However, in addition to presynaptic expression of synaptic long term plasticity (Nicoll and Schmitz, 2005), slow homeostatic regulations occur in the presynapse, e.g., in form of chronic receptor or ion channel modulations.

### Presynaptic ion channel and glycine receptor plasticity

**Figure 1:**
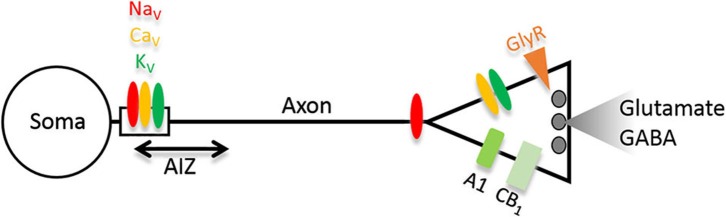
**Scheme depicting strategic molecules relevant for homeostasis of presynaptic function and epilepsy discussed in this minireview**. In glutamatergic neurons, gain-of-function of molecules colored red/orange will increase network excitability, whereas those colored green will decrease it. The opposite holds true if the changes occur in GABAergic neurons. Abbreviations: Na*_V_*, Ca*_V_*, K*_V_*, voltage-gated sodium, calcium and potassium channels; AIZ, action potential (AP) initiation zone; GlyR, glycine receptors; A1, adenosine receptor; CB_1_, cannabinoid receptor 1.

Because transmitter release is controlled by action potential-(AP)-triggered calcium influx in the synaptic terminal, regulation of ion channels which shape the axonal AP and terminal depolarization is an effective mechanism of presynaptic plasticity. With this definition, the AP initiation zone (AIZ) could be viewed as part of the presynaptic equipment. A striking form of homeostatic plasticity has been documented for the AIZ: this entire subcellular structure including voltage-gated Na^+^ (Na*_v_*) and K^+^ (K*_v_*) channels can be shifted along the axon (Figure 1), thereby counteracting hyperexcitation by increasing thresholds for AP generation (Grubb and Burrone, 2010). Such axonal remodeling may be facilitated via ion channel trafficking regulated by alternative splicing, as shown for “shaw-related” Kv3 channels (Gu et al., 2012). Other important constituents of presynaptic control are “shaker-related” K*_V_*1 channels (Wang et al., 1994). Their role is well demonstrated for the large glutamatergic mossy fiber boutons of dentate granule cells, which impinge on hippocampal CA3 pyramidal cells (Geiger and Jonas, 2000; Bischofberger et al., 2006). Here, K*_v_* channels could gain further importance during temporal lobe epilepsy (TLE), when seizures invade the hippocampus and feedforward inhibition of CA3 pyramidal cells via interneurons is compromised (Lawrence and McBain, 2003). Indeed, seizures trigger a transcriptional upregulation of K*_v_*1.1 channels in granule cells, thereby delaying their AP responses considerably, as recently shown in a TLE mouse model (Kirchheim et al., 2013). Consistent with the view that K*_V_*1.1 is a promising antiepileptic target, K*_V_*1.1 knockout mice develop epilepsy (Wenzel et al., 2007) and lentiviral overexpression of K*_V_*1.1 ameliorates seizures in an animal model of neocortical epilepsy (Wykes et al., 2012). The interaction of activity-dependent downscaling and potentiation of presynaptic excitability may involve the adenylyl cyclase pathway (Nicoll and Schmitz, 2005) but it is still unclear how exactly these seemingly opposed mechanisms interact in the same presynaptic compartment.

Epilepsy often comes with cognitive dysfunction and neuropsychiatric comorbidities (García-Morales et al., 2008). We discovered a molecule which in this regard may have an important impact: an RNA variant of the neurotransmitter receptor for glycine (GlyR). The GlyRs are subject to increased RNA editing in resected hippocampi of TLE patients (Eichler et al., 2008) which profoundly influences biophysical receptor properties. The reason is an amino acid substitution in the ligand binding domain leading to gain-of-function receptors with increased neurotransmitter affinity (Meier et al., 2005; Eichler et al., 2008; Legendre et al., 2009) and spontaneous channel activity (Kletke et al., 2013; Winkelmann et al., 2014). In addition, RNA splicing governs presynaptic GlyR expression (Winkelmann et al., 2014), and in hippocampal neurons, the lack of the GlyR β subunit (Weltzien et al., 2012) which governs postsynaptic receptor clustering (Meyer et al., 1995; Meier et al., 2000, 2001; Eichler et al., 2009; Förstera et al., 2010; Kowalczyk et al., 2013), certainly facilitates GlyR expression and function at presynapses (Figure 1). Presynaptic GlyRs are tightly packed (~200 receptor channels in a cluster with ~100 nm radius; Notelaers et al., 2012, 2014a,b), which implies that a single presynaptic cluster from the spontaneously active GlyR RNA variant will have a considerable functional impact on synaptic neurotransmitter release, even if the contribution of the glycinergic system to this brain region appears limited (Zeilhofer et al., 2005). Consistent with the excitatory nature of presynaptic chloride channels and the well documented presynaptic GlyR expression in the hippocampus (Kubota et al., 2010; Ruiz et al., 2010; Waseem and Fedorovich, 2010; Winkelmann et al., 2014), we found that the spontaneously active GlyR RNA variant actually increased presynaptic excitability and the functional impact of glutamatergic neurons or parvalbumin-positive interneurons* in vivo* and, depending on the type of neuron, triggered cognitive dysfunction or anxiety in our mouse model of epilepsy (Winkelmann et al., 2014). In agreement with the proposed critical role of presynaptic GlyRs in the regulation of neural network excitability, application of a low, non-receptor-saturating, glycine concentration (10 µM) to corticohippocampal slice preparations was sufficient to enhance epileptiform activity induced by block of K_V_1 channels (Chen et al., 2014).

### Retrograde autocrine and paracrine signaling

Although the idea of cannabis as a potential antiepileptic drug is ancient, it remained elusive how it could work reliably (Adams and Martin, 1996; Miller, 2013). Recent discoveries on endogenous cannabinoid receptors (CB), of which particularly CB_1_ is widely expressed in presynaptic terminals of excitatory and inhibitory neurons (Figure 1), could lead to a better understanding of CB mechanisms in epilepsy (Alger, 2004; Katona and Freund, 2008; Hill et al., 2012). In GABAergic neurons, activation of CB_1_, e.g., via neuronal activity-dependent retrograde post-to-presynapse release of CBs anandamide or 2-AG has been shown to decrease synaptic GABA release, a mechanism termed depolarization-induced suppression of inhibition (DSI; Ohno-Shosaku et al., 2001; Wilson et al., 2001). Consistently, elevated CB_1_ presence observed in epilepsy models and TLE patients (Goffin et al., 2011; Karlócai et al., 2011; Bojnik et al., 2012) has been interpreted as proconvulsive (Chen et al., 2003, 2007). On the other hand, CB_1_ is also expressed on glutamatergic terminals, where its activation reduces glutamate release (Domenici et al., 2006; Kawamura et al., 2006). Furthermore, CB_1_ activation increases inward rectifier K^+^ (Kir) currents (Mackie et al., 1995; Chemin et al., 2001) mediated via postsynaptic channels which are also upregulated in TLE (Young et al., 2009; Stegen et al., 2012). In summary, while elevation of CB_1_ at GABAergic synapses and reduction at glutamatergic synapses likely constitute endogenous adaptations to epilepsy, exogenous CB_1_ overexpression and activation in principal neurons, possibly via receptors physiologically rarely activated, could effectively protect against seizures (Blair et al., 2006; Guggenhuber et al., 2010; Hofmann and Frazier, 2013).

Adenosine triphosphate (ATP) is released from astrocytes and can enhance neuronal excitability through its direct action onto purinergic receptors. However, ATP can also exert indirect effects upon its enzymatic conversion to adenosine and signaling through adenosine A1 receptors, which reduces synaptic glutamate release (Nicoll and Schmitz, 2005; Boison, 2013; Dias et al., 2013). Therefore, adenosine signaling is another mechanism of presynaptic homeostasis with recognized relevance to epilepsy; while too much adenosine clearance via gliosis-enhanced adenosine kinase activity is a proconvulsive factor, adenosine augmentation in the epileptic focus represents a powerful anticonvulsive principle (Boison, 2012).

### Perspective

In agreement with the proposed critical role of presynaptic compartments in the regulation of neural network homeostasis, diverse pharmacological agents with a presynaptic mode of action were reported to be effective in the treatment of epilepsy. In particular drugs which provide rapid adaptation against excessive excitation, e.g., via use-dependent inhibition of Na*_v_* or Ca*_v_* channels (phenytoin, carbamazepine, lamotrigine, topiramate, and levetiracetam), likely act primarily in axons (Stefani et al., 1996; Wu et al., 1998; Catterall, 1999; Vogl et al., 2012). Interestingly, these drugs also have effects on psychiatric symptoms (Barbosa et al., 2003; Lexi-Comp, 2009; Andrus and Gilbert, 2010) indicating common underlying mechanisms of cognitive dysfunction and psychiatric symptoms of epilepsy. With more research on neuron type-specific roles in behavior (Lovett-Barron et al., 2014; Winkelmann et al., 2014), new antiepileptic strategies could ground on these insights and specifically target presynaptic molecules in the affected cell types.

## Conflict of interest statement

The authors declare that the research was conducted in the absence of any commercial or financial relationships that could be construed as a potential conflict of interest.
