# Erratum on: Presynaptic mechanisms of neuronal plasticity and their role in epilepsy

**DOI:** 10.3389/fncel.2014.00206

**Published:** 2014-08-04

**Authors:** Frontiers Production Office

**Affiliations:** Frontiers Production Office, FrontiersLausanne, Switzerland

**Keywords:** epilepsy, axon, RNA editing, potassium channels, glycine receptor, homeostatic regulation, neuropsychiatric disorders, hippocampus

Due to a production error, in the published article the middle initial for the first author Jochen C. Meier was removed. This error does not change the scientific conclusions of the article in any way. The publisher apologizes for this mistake.

The original article has been updated.

## Conflict of interest statement

The authors declare that the research was conducted in the absence of any commercial or financial relationships that could be construed as a potential conflict of interest.
